# Advances in the Mass Sensitivity Distribution of Quartz Crystal Microbalances: A Review

**DOI:** 10.3390/s22145112

**Published:** 2022-07-07

**Authors:** Xianhe Huang, Qiao Chen, Wei Pan, Yao Yao

**Affiliations:** School of Automation Engineering, University of Electronic Science and Technology of China, Chengdu 611731, China; qiaochen@std.uestc.edu.cn (Q.C.); weipan@std.uestc.edu.cn (W.P.); yaoyao428@uestc.edu.cn (Y.Y.)

**Keywords:** quartz crystal microbalance (QCM), mass sensitivity, electrode parameter, particle displacement amplitude

## Abstract

A quartz crystal microbalance (QCM) is a typical acoustic transducer that undergoes a frequency shift due to changes in the mass of its surface. Its high sensitivity, robustness, small size design, and digital output have led to its widespread development for application in the fields of chemistry, physics, biology, medicine, and surface science. Mass sensitivity is one of the vital parameters and forms the basis for quantitative analysis using QCMs. This review firstly introduces the importance, definition, calculation, and measuring method of the mass sensitivity and then focuses on reviewing the influence of electrode parameters (including electrode shape, electrode diameter, electrode thickness, electrode material, etc.) on the mass sensitivity distribution of QCMs. Finally, the effect of the operating frequency on the mass sensitivity of QCMs is also analyzed.

## 1. Introduction

A quartz crystal microbalance (QCM) is a typical bulk acoustic wave device that consists of a vibrating quartz plate sandwiched between two metal excitation electrodes. Due to the piezoelectric effect of quartz crystals, the QCM is a typical mass sensor that can translate the mass change on its surface into a frequency change [1,2,3]. With the advantages of simple structure, easy operation, low cost, high sensitivity, and measurement accuracy that can reach the nanogram level, QCMs have been widely used in chemistry, physics, biology, medicine, and surface science for compositional analysis of gases and liquids, as well as measurement of micromasses and film thickness and in viscoelastic structure detection [4,5,6,7,8,9,10]. The further development of the subsequent electrochemical quartz crystal microbalance (E-QCM) and quartz crystal microbalance with dissipation (QCM-D) techniques facilitate online tracking to detect changes in microscopic processes, with the advantage of access to rich information that is not possible with other methods [11,12,13,14,15,16,17,18,19,20,21]. 

A large number of review articles of QCM-related works have been published in the past. However, they are mainly application-oriented review articles. For example., Thompson et al. reviewed the theoretical aspects of QCMs for liquid phase operation and suggested measurement methods and applications [22]. Fauzi et al. focused on the graphene-material-based QCM sensors for gas and humidity detection [6]. Mujahid et al. provided a comparative review of high-frequency acoustic sensors (mainly QCMs, SAWs, and FBARs) for chemical and biochemical applications [23]. Later, Dirri et al. provided a review of QCM sensors for monitoring contaminants in space missions, i.e., space shuttle flights, NASA Space Transportation System (NASA STS), and satellite missions [24]. Akgöneüllü et al. reviewed QCM biosensors based on the molecular imprinting technique for disease-related biomarkers [25]. In addition, Alassi et al. presented a comprehensive review of the various existing electronic interfacing systems of QCMs, including impedance-based analysis, oscillators (conventional and lock-in based techniques), exponential decay methods, and the emerging phase-mass-based characterization [26]. However, we note that no articles have reviewed the definition, theory, measuring methods, and influence of electrode parameters on the mass sensitivity of QCMs, which was the original intention of this review.

The core component of the QCM is a quartz crystal resonator, so early publications on this topic also referred to it directly as a QCR, which was first used as a frequency control element [27,28,29]. As early as 1944, Bottom pointed out that the frequency change of a quartz crystal resonator during the aging process is proportional to the change in its surface thickness [30]. Around 15 years later, Sauerbrey theoretically deduced the relationship between the frequency shift and the mass change on the surface of QCM when ignoring the effect of the metal electrodes as [2,31]

(1) $$\Delta f=-\frac{2f_{0}^{2}\Delta m}{A\sqrt{\rho_{q}\mu_{q}}}=-C_{f}\cdot \Delta m$$

where the negative sign indicates that the resonant frequency decreases as the additional mass on the surface of QCM increases. $f_{0}$ is the fundamental frequency of the QCM, $\rho_{q}$ (=2.648 g/cm^3^) is the quartz crystal density, $\mu_{q}$ (=2.947 × 10^11^ g/cm·s^2^) is the shear modulus of the AT-cut quartz crystal, A is the effective vibration area, and $\;C_{f}$ is the Sauerbrey mass sensitivity. Δm and Δf are the additional mass attached to the surface of QCM and the corresponding frequency shift, respectively. However, it is worth noting that the Sauerbrey equation is satisfied when the additional mass layer is rigid, uniformly distributed, and of small mass. The Sauerbrey equation can be used to quantify the relationship between mass information and electrical signals, thus laying the theoretical foundation for the use of a QCM as a mass sensor.

However, in practice, QCMs must have metal electrodes, so the effect of metal electrodes on the mass sensitivity cannot be ignored [32,33]. Due to the metal electrodes, the QCM mass sensitivity cannot simply be considered as a constant, but as a Gaussian distribution [34,35]. The electrode parameters have great influence on the mass sensitivity of the QCM, which may lead to increased errors in quantitative analysis. In quantitative analysis in the gas phase, $C_{f}$—which is the integration of the mass sensitivity over the sensing area, also known as the equivalent mass sensitivity—is mainly used, so the mass sensitivity distribution is important for QCM gas phase applications [31]. 

For a single drop of liquid on the center of a QCM electrode, the frequency change can be calculated using the following equation [36,37]:

(2) $$\Delta f=-\frac{1}{2}Kr_{a}^{2}\sqrt{\frac{\pi \rho_{L}\eta_{L}}{f_{0}}}\left(1+\frac{\beta r_{d}^{2}}{2r_{e}^{2}}\right)$$

where $r_{e}$ and $r_{d}$ are the radii of the electrode and droplet, respectively. $\rho_{L}$ and $\mu_{L}$ are the density and viscosity of the liquid, respectively. K is also the equivalent mass sensitivity, which means that the QCM mass sensitivity plays a decisive role in the frequency response of a single drop of liquid. It can be seen that the frequency variation of the QCM is still proportional to the mass sensitivity distribution in the droplet test. 

Kanazawa et al. derived the formula for calculating the frequency change of the QCM when one surface of QCM is fully immersed in a Newtonian liquid [13,14]:

(3) $$\Delta f=f_{0}^{3/2}{\left(\frac{\rho_{L}\eta_{L}}{\pi \rho_{q}\mu_{q}}\right)}^{1/2}$$

The thickness shear wave of the QCM is transmitted into the liquid interior through the contact with the liquid surface, and this shear wave rapidly decays to zero under the damping effect of the liquid load. According to acoustic wave theory, the penetration depth of the acoustic wave in the liquid is determined by the acoustic frequency, liquid viscosity, and liquid density [36].

(4) $$\delta =\sqrt{\frac{\eta_{L}}{\pi \rho_{L}f_{0}}}$$

Only the liquid layer within the depth of acoustic wave penetration, also known as the effective mass layer, affects the resonance frequency of the QCM [38]. Therefore, it can be shown that Equation (3) can be exactly equated to Equation (1). That is, the frequency change of Kanazawa’s equation in the liquid phase test is still proportional to the equivalent mass sensitivity, which means that mass sensitivity has an indispensable effect on the quantitative analysis of QCM in both gas and liquid phases. Therefore, it is necessary to investigate the influence of electrode parameters (electrode shape, electrode diameter, electrode thickness, electrode material, etc.) on the mass sensitivity distribution of QCM.

## 2. Theory and Measuring Method

The equivalent mass sensitivity, $C_{QCM}$, is defined as [31]

(5) $$C_{QCM}=\frac{1}{\pi r_{s}^{2}}\int_{0}^{r_{s}}2\pi rS_{f}\left(r\right)dr$$

where $r_{s}$ is the radius of the mass load attached on the QCM. $S_{f}\left(r,\theta \right)$ is the mass sensitivity function distribution of QCMs, and is defined by [39]

(6) $$S_{f}\left(r,\theta \right)=\frac{\left|\tilde{u}\left(r,\theta \right)\right|}{\int_{0}^{\infty }\int_{0}^{2\pi }r|\tilde{u}\left(r,\theta \right){|}^{2}d\theta dr}C_{f}$$

where $\tilde{u}\left(r,\theta \right)$ is the particle displacement amplitude. r and θ are the radius and angle of the specified point in polar coordinates, respectively. The particle displacement amplitude $\tilde{u}\left(r,\theta \right)$ and direction *θ* can be considered to be independent, and the above equation can thus be simplified as [40]

(7) $$S_{f}\left(r\right)=\frac{\left|\tilde{u}\left(r\right)\right|}{2\pi \int_{0}^{\infty }r|\tilde{u}\left(r\right){|}^{2}dr}C_{f}$$

The denominator in Equation (7) is the intensity of the total particle displacement amplitude of the QCM, which is a constant after integration. $\tilde{u}\left(r\right)$ can be evaluate by the following Bessel equation.

(8) $$r^{2}\frac{\partial^{2}{\tilde{u}}_{r}}{\partial r^{2}}+\frac{\partial {\tilde{u}}_{r}}{\partial r}+\frac{k_{i}^{2}r^{2}}{N}{\tilde{u}}_{r}=0$$

where N is determined by the material constants of the quartz crystal; $k_{i}^{2}=\left(\omega^{2}-\omega_{i}^{2}\right)/c^{2}$, in which i = *E*, *P*, *U* (*E*, *P*, and *U* refer to the fully electroded region, partially electroded region, and non-electroded region, respectively); $c=\sqrt{c_{66}/\rho_{q}}$ is the acoustic wave velocity in the quartz crystal (where $c_{66}$ is the elastic stiffness constant); and $\omega_{i}$ (i = *E*, *P*, *U*) is the cut-off frequency of each region.

It is worth noting that the mass sensitivity distribution of the QCM is related to the particle displacement amplitude function and the distance from a given point to the electrode center, making it difficult to directly measure the mass sensitivity. For this reason, pioneers have used various methods to observe the trends of the mass sensitivity distribution to validate their proposed model [15,34,35,41]. In 1989, Martin and Hager measured the variation of the series resonant frequency of the AT-cut QCM when a fine tungsten wire was pulled across the surface of a QCM [15]. It was found that the frequency change was biggest when the fine tungsten wire was pulled near the center of the QCM surface, indicating that the mass sensitivity was maximum at the center of the electrode surface. Using these results, they attempted to relate the resulting frequency change to the vibration amplitude or mass sensitivity of the piezoelectric crystal resonator. Later, Ward, Hillier, and Richardson et al. used the ink dot method to test the mass sensitivity distribution function of the QCM [34,35,41], wherein an ink dot from a fine-tipped felt pen were placed in precise radial positions, and the resulting frequency shifts were recorded after the drops had completely dried. In 2009, Kawasaki et al. [42] found a reproducible phenomenon by which laser irradiation onto the QCM surface resulting in an increase in its frequency. Therefore, they used weak laser irradiation (523–785 nm, 5–60 mW) onto the gold electrode surface of a 27 MHz QCM to test the dependence of the frequency change with respect to the irradiation position. The results also verify the Gaussian distribution of the QCM mass sensitivity.

**Figure 1:**
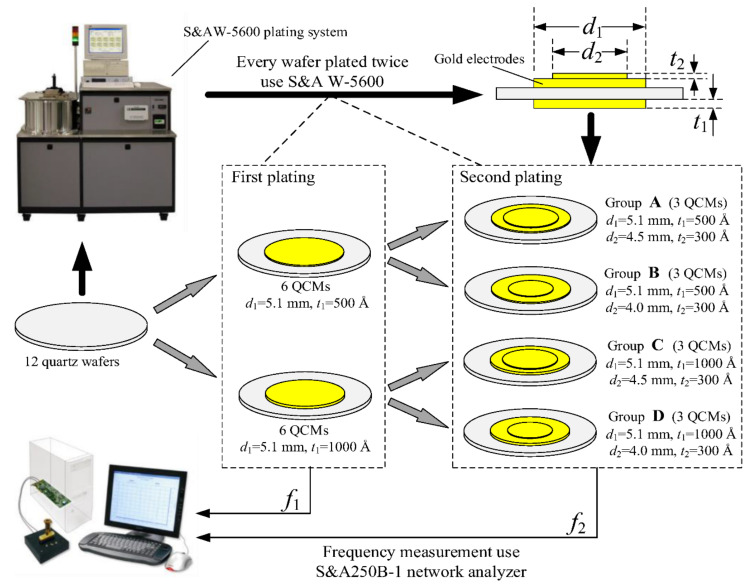
Schematic diagram of the experimental setup for testing the mass sensitivity of a QCM using vacuum coating method [31].

However, in using the ink dot method, it is actually difficult to satisfy the conditions of the Sauerbrey equation that the small mass layer is uniformly and rigidly adsorbed onto the QCM surface, while mass sensitivity cannot be directly obtained using the laser method. In 2017, Huang et al. proposed a method to measure the mass sensitivity of QCMs by vacuum coating, and the schematic diagram of the experimental setup is shown in Figure 1 [31]. The procedure is as follows: Firstly, metal electrodes of different parameters (size, material, shape, thickness, etc.) are vaporized on the surface of bare quartz crystal using different shapes and sizes of masks to prepare QCMs with different electrode parameters, and a second plating is then applied according to the requirements of experiments (e.g., different diameters and thicknesses). The frequencies and equivalent parameters before and after the second plating are then measured using S&A250B-1 network analyzer. Finally, the mass sensitivities of the QCM can be calculated from the mass and frequency changes before and after the second coating. This method not only allows constructing metal electrodes with different parameters (e.g., diameter, shape, thickness, material, etc.) but also fully satisfies the three conditions of the Sauerbrey equation. In addition, Gabrielli, and Hu et al. used electrochemical deposition coating to verify the mass sensitivity of the QCM [43,44,45]. 

## 3. Electrode Shape and Mass Sensitivity

**Figure 2:**
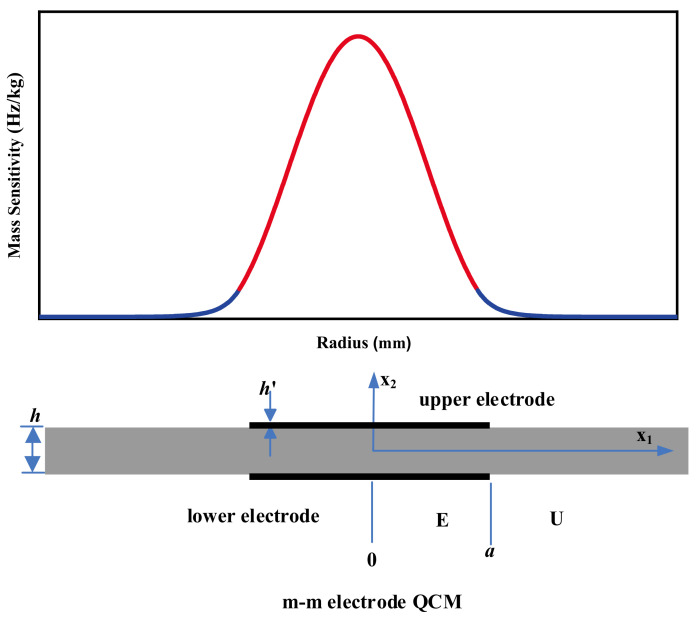
Schematic diagram of the structure and mass sensitivity distribution of the m-m electrode QCM [31]. Here *a* is the radius of the circular metal electrode. h and $h^{\prime }$ are the thickness of the quartz wafer and the metal electrode, respectively.

The shape commonly used for QCM electrodes is a symmetric circular configuration, also known as the m-m electrode QCM, as shown in Figure 2. Many scholars including Cumpson, Hillier, and Josse, have calculated the mass sensitivity distribution of the m-m electrode QCMs and found that it conforms to Gaussian distribution [34,35,39,46]. Cumpson et al. found that the higher the electrode density, the more the QCM vibration is confined to the electrode center [46].

**Figure 3:**
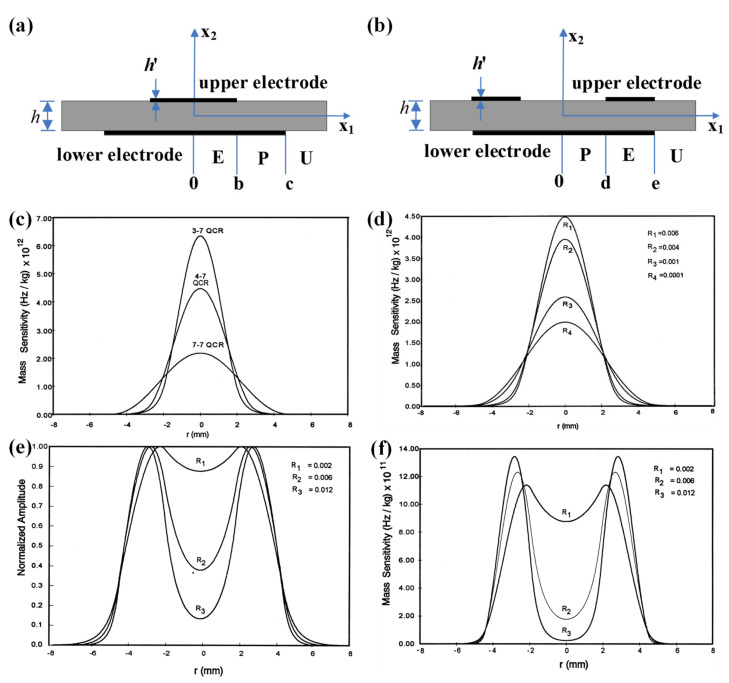
Schematic diagram of the structure of the (**a**) n-m electrode QCM and (**b**) ring electrode QCM. The mass sensitivity distribution function of n-m electrode 11 MHz AT-cut QCMs under (**c**) different diameters and (**d**) different mass loading factors. (**e**) Particle displacement amplitude profile and (**f**) mass sensitivity distribution function of ring electrode 11 MHz AT-cut QCMs [39]. b and c are the radii of the upper electrode and lower electrode for the n-m electrode QCM, respectively. d and e are the radii of the inner and outer electrode, respectively.

In 1998, Josse et al. were the first to analyze the radial dependence of the mass sensitivity distribution for QCMs with modified electrode shapes, mainly n-m electrode QCMs and ring electrode QCMs, as shown in Figure 3a,b, respectively [39]. The n-m electrode QCM has circular electrodes on both upper and lower sides, but the diameter of the upper electrode (called n electrode) is smaller than that of the lower electrode (called m electrode). Generally, the diameter of the upper and lower electrodes are directly used to name the n-m electrode QCM; for example, the name 4–7 QCM indicates that the upper and lower diameters of the n-m electrode QCM are divided into 4 and 7 mm, respectively [39]. In the field of frequency control, it is recognized that quartz crystal resonators with n-m electrodes have a higher Q value than quartz crystal resonators with m-m electrodes, which means better stability. Josse et al. concluded that compared with the conventional m-m electrode QCM, the energy is more concentrated in the central region of the smaller electrode, which therefore have a higher mass sensitivity. The calculated results for the mass sensitivity distribution of the AT-cut, 11 MHz QCM with a mass loading factor of 0.006, are shown in Figure 3c. The 4–7 QCM has a significantly higher sensitivity curve than 7–7 QCM, which indicates that the mass sensitivity distribution of the n-m electrode QCM is significantly higher than that of the m-m electrode QCM. The 3–7 QCM has a significantly higher sensitivity curve than 4–7 QCM, which indicates that the smaller the electrode, the more energy concentrated at the center of the QCM and, therefore, the higher mass sensitivity. They also calculated the mass sensitivity of QCM with different mass loading factors for 4–7 QCMs, as shown in Figure 3d. It can be seen from the results that the larger the mass loading factor, the greater the mass sensitivity of n-m electrode QCM. The ring electrode QCM, as shown in Figure 3b, has a ring electrode on the upper surface and a circular electrode on the lower surface. Figure 3e,f show the particle displacement amplitude and mass sensitivity function distribution of the ring electrode QCM, respectively. It can be seen that the mass sensitivity of the ring electrode QCM shows a bimodal distribution, rather than the Gaussian-type mass sensitivity function distribution of the m-m electrode QCM and n-m electrode QCM, and the concavity trend between the bimodal peaks can be optimized by adjusting the mass loading factor.

The Gaussian-type mass sensitivity distribution is not conducive to the quantitative analysis of QCM in practical applications, which is one of the reasons for poor reproducibility of QCM quantitative analysis [47]. Therefore, scholars hope to obtain uniform mass sensitivity by optimizing the QCM electrode shape, i.e., the mass sensitivity becomes a constant in the central region. In 2008, Richardson et al. continued to explore the mass sensitivity distribution of ring electrode QCM in depth with Josse’s work [41,48]. They illustrated, by calculation, that uniform mass sensitivity distribution can be obtained for AT-cut ring electrode with inner and outer diameters of 4 and 10 mm, respectively, at a mass loading factor of 0.0025. They tested the mass sensitivity distribution of this ring electrode QCM using the droplet weight method. Unfortunately, the experimental results showed that there is still a concavity between the double peaks of the mass sensitivity distribution of this ring electrode QCM.

Shi et al. investigated the mass sensitivity distribution of an AT-cut rectangular ring electrode QCM [49]. The electrode was also rectangular with its central part missing, thus forming a rectangular ring electrode. It was found that the vibrations tend to be trapped in the electrode region and decay away from the edge of the electrode. Through theoretical calculations and finite element analysis, the authors also demonstrated that by properly designing the electrode size, the vibrations in the central part of the plate can be made almost uniform, resulting in uniform mass sensitivity distribution. Unfortunately, there is no experimental evidence to support their idea.

**Figure 4:**
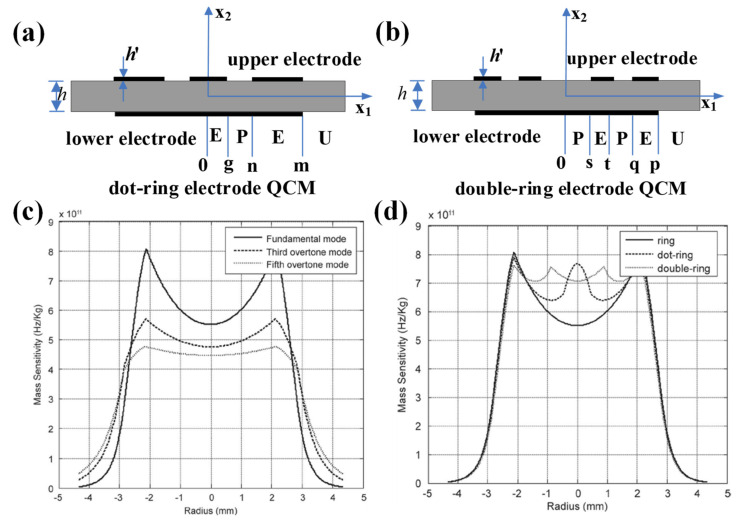
Schematic diagram of the structure of the (**a**) dot-ring electrode QCM and (**b**) double-ring electrode QCM. Mass sensitivity distribution for (**c**) ring electrode QCMs with fundamental, third overtone, and fifth overtone operating frequencies of 10 MHz. (**d**) Ring, dot-ring, and double-ring electrode quartz crystal resonators with fundamental operating frequencies of 10 MHz [50]. Here, g, n, and m denote the radius of the center dot and the inner and outer radii of the outer ring for the dot-ring electrode QCM, respectively. s, t, q, and p denote the inner and outer radii of the inner ring and the inner and outer radii of the outer ring for the double ring electrode QCM, respectively.

In 2013, Gao et al. investigated the mass sensitivity distributions of several other modified electrode QCMs, mainly ring electrode QCM, dot-ring electrode QCM, and double-ring electrode QCM, in order to obtain uniform mass sensitivity function distribution [50]. The thicknesses of quartz wafer for fundamental, third overtone, and fifth overtone operating frequencies of 10 MHz QCM were 0.1648, 0.4974, and 0.8298 mm, respectively, with an electrode thickness of 1000 Å and inner and outer diameters of 2.12 and 2.85 mm, respectively. They first calculated the mass sensitivity distributions for fundamental, third overtone, and fifth overtone operating frequencies of 10 MHz, as shown in Figure 4c, and it can be seen that the fifth overtone mode is more suitable for obtaining uniform mass sensitivity function distribution. Then, the authors presented the dot-ring electrode QCM, and the double-ring electrode QCM, as shown in Figure 4a,b, respectively. Figure 4d shows the theoretical mass sensitivity function distributions calculated by authors. It can be seen that by forming two additional peak points, the concavity of the mass sensitivity distribution for the double-ring electrode QCM is significantly reduced compared with the ring electrode QCM and the dot-ring electrode QCM. It is worth noting that the mass sensitivity curve of QCM should be smooth and derivable everywhere, so there is room for further refinement of the calculation by Gao et al.

**Figure 5:**
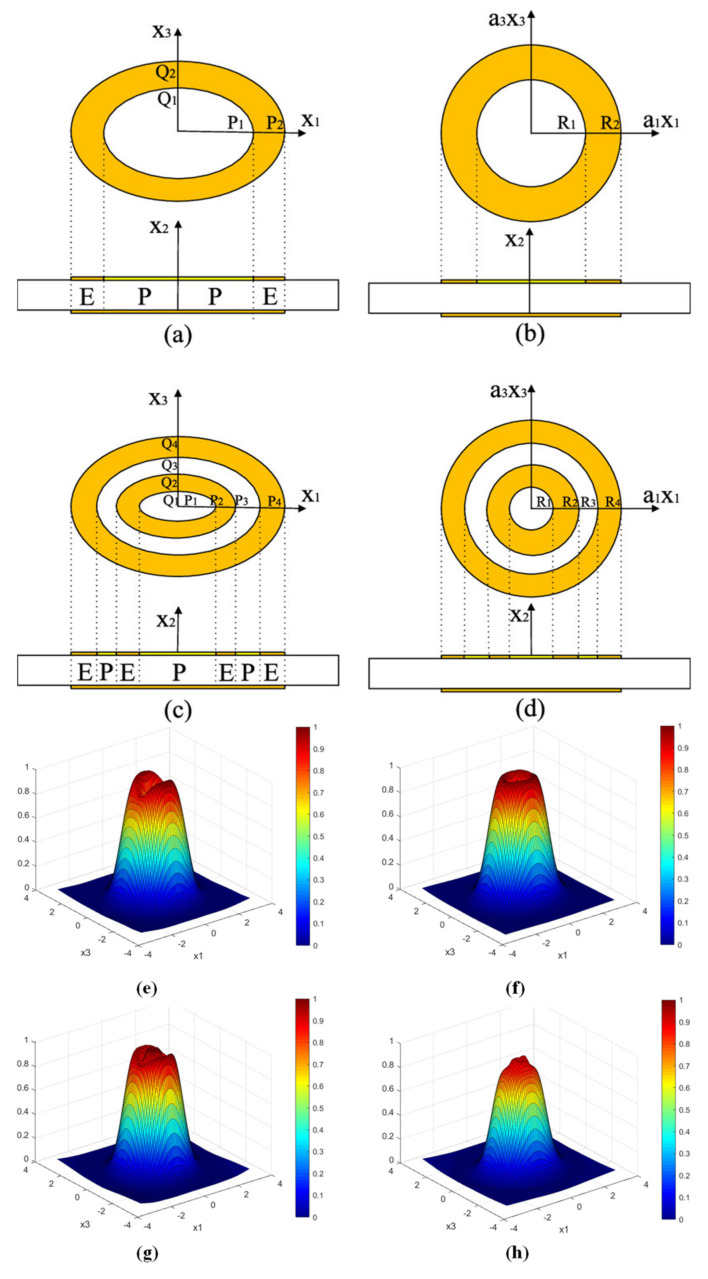
Structure of QCM with (**a**) elliptical single ring, (**b**) circular ring, (**c**) elliptical double ring, and (**d**) circular double ring electrodes. R_1_, R_2_, R_3_, and R_4_ are the radii of each circular electrode, respectively, and P_1_, P_2_, P_3_, and P_4_ are the major semi-axes of each elliptical electrode, respectively. Q_1_, Q_2_, Q_3_, and Q_4_ are the short semi-axes of each elliptical electrode, respectively. Surface displacement distribution of (**e**) circular single-ring electrode QCM, (**f**) elliptical single-ring electrode QCM, (**g**) circular double-ring electrode QCM, and (**h**) elliptical double-ring electrode QCM [53].

Based on Gao et al., other additional configurations of electrode shapes have been designed to obtain uniform mass sensitivity distribution [49,51,52,53]. For example, Jiang et al. used mathematical models and the finite element method (FEM) to further analyze in depth the mass sensitivity function distribution of the double-ring electrode QCM [52]. Through theoretical calculations and finite element simulations, they found that the mass sensitivity distribution of the double-ring electrode QCM varies with the electrode sizes s, t, n, and m. The average mass sensitivity of the double-ring electrode QCM was at its maximum and most uniformly distributed in this simulation when the size of s is close to half of n. This conclusion also applies when the value of t is close to half of m. Moreover, when the size of the electroded region is larger than the size of the partial electroded region (within the limit), the average mass sensitivity value will increase. In addition, there exists an optimal electrode outer radius with a more uniform displacement distribution for the double-ring electrode QCM. Soon after, they continued to investigate the distribution of the uniform mass sensitivity function of the ellipsoidal electrode QCM, as shown in Figure 5 [53]. Their results show that in order to achieve the uniformity of the mass sensitivity of the elliptical ring electrode QCM, it is recommended that the ratio of the short axis to the long axis of the elliptical ring electrode should be 0.8. In addition, by selecting the appropriate parameters, a more uniform mass sensitivity function can be obtained for the elliptical double ring electrode QCMs.

**Figure 6:**
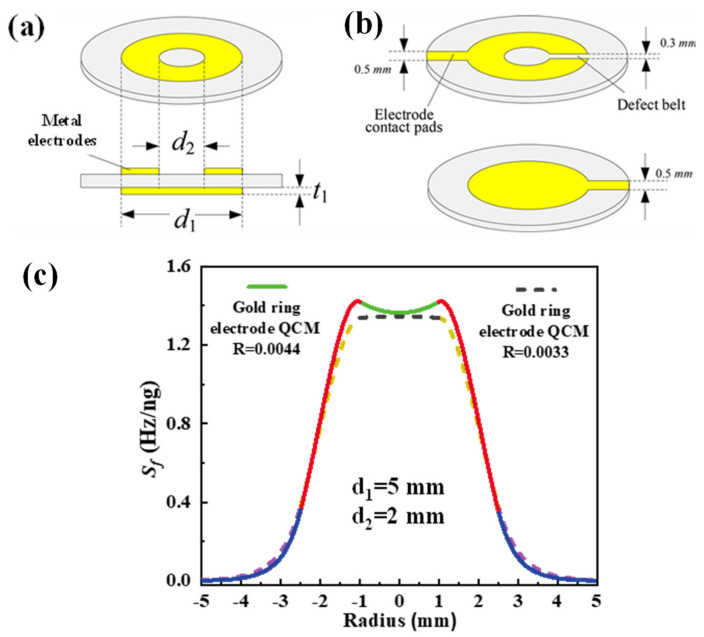
(**a**) The angled view (upper) and profile map (lower) of the ideal ring electrode QCM, where *t*_1_ is the thickness of the electrode and *d*_1_ and *d*_2_ are the outer and inner diameters of electrode, respectively. (**b**) The ring-electrode on the topside (upper) and circle-electrode on the backside (lower) of actual ring electrode QCM. (**c**) Mass sensitivity distribution of AT-cut 10 MHz ring electrode QCM having inner and outer diameters of 2 and 5 mm, respectively [54,55].

However, the mentioned electrode shapes are too complicated to process and are not conducive to practical use. Therefore, the mass sensitivity distributions of these QCMs with different electrode shapes have not been experimentally verified. In 2018, through theoretical calculation analysis, Huang et al. found that beyond the electrode mass loading factor, the inner and outer diameters of the ring electrode also have a significant effect on the mass sensitivity distribution of the ring electrode QCMs [47,54,55,56]. They concluded that uniform mass sensitivity distribution can be obtained with a ring electrode QCM when satisfying conditions of a suitable (1) ratio of inner and outer diameters; (2) inner diameter value; and (3) electrode thickness (depending on the mass loading factor). Take the AT-cut 10 MHz ring electrode QCM with inner and outer diameters of 2 and 5 mm, respectively, as an example, shown in Figure 6a. When the electrode material was gold, a mass loading factor of 0.0044 was suggested to obtain uniform mass sensitivity. For a silver electrode, the mass loading factor should be 0.0033. The theoretical calculation results are shown in Figure 6b. Finally, they demonstrated that mass sensitivity distribution was approximately uniform using the coating experiment.

## 4. Electrode Diameter (Area) and Mass Sensitivity

**Figure 7:**
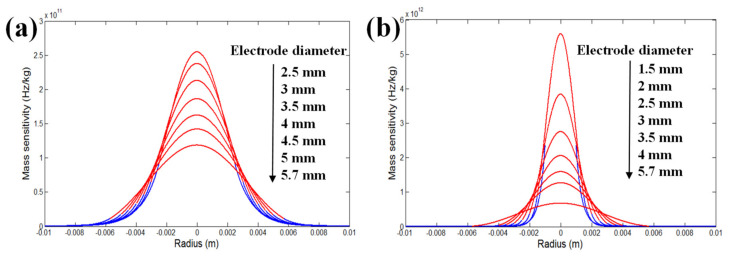
Mass sensitivity distribution profiles for AT-cut (**a**) 5 MHz and (**b**) 10 MHz QCMs with different electrode diameters [31].

The electrode diameter (area) greatly affects the vibrational amplitude of the thickness shear mode such that it is largely concentrated in the central region of the QCM due to the energy trapping effect, which in turn affects the mass sensitivity distribution of the QCM [43]. In 2017, Huang et al. calculated the mass sensitivity distributions of the m-m QCMs with AT-cut and fundamental frequencies of 5 and 10 MHz, respectively, for different electrode diameters, as shown in Figure 7 [31]. The thicknesses of the gold electrodes were both 1000 Å. The thicknesses of the QCMs at 5 and 10 MHz were 333 and 167 µm, respectively. The red line region is the electroded region and the blue line region is the non-electroded region. It can be seen that for these circular m-m QCMs, the maximum mass sensitivity point is located at the center of the circular electrode and decays exponentially with increasing radial distance from the center. However, it is worth noting that for QCMs with the same resonant frequency but different electrode diameters, their mass sensitivity distributions are extremely different. Then, through the theoretical calculations, it is found that Sauerbrey’s mass sensitivity does not accurately reflect the frequency versus mass loading of the QCM, especially for the case of small electrodes or of large electrodes loaded with small loads. Therefore, Huang et al. proposed an equivalent mass sensitivity model that considers both the Gaussian distribution properties of the mass sensitivity and the influence of the electrodes on the mass sensitivity distribution. Finally, the correctness of the model was confirmed using a series of coating experiments. 

**Figure 8:**
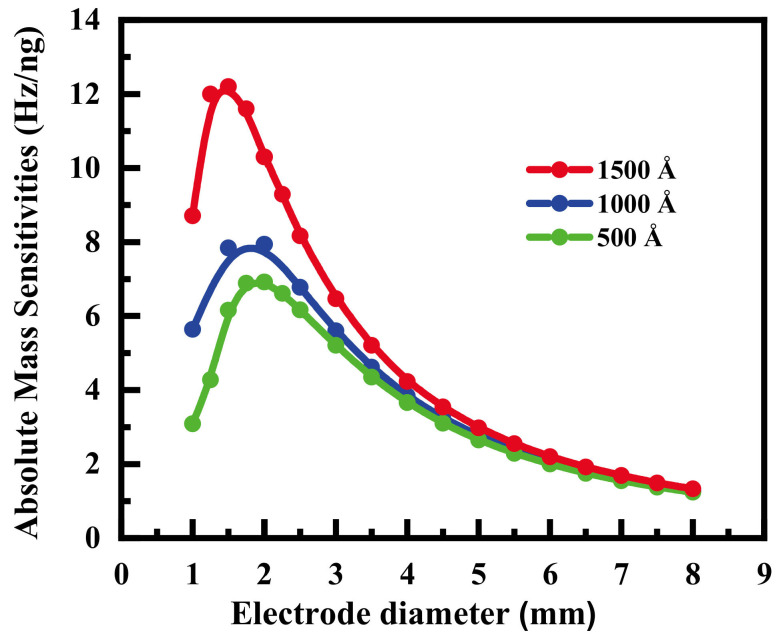
Theoretical absolute mass sensitivity distribution of AT-cut 10 MHz QCM with different electrode diameters and electrode thicknesses [57,58].

Next, Huang’s team continued to investigate the effect of electrode diameter on the m-m electrode QCM mass sensitivity distribution [57,58,59]. They first calculated the absolute mass sensitivity (i.e., the mass sensitivity at the center of the QCM) of the AT-cut, 10 MHz m-m QCM for different electrode diameters and different electrode thicknesses, as shown in Figure 8. It can be seen from the figure that the absolute mass sensitivity does not vary monotonically with the electrode diameter but that a maximum point exists. In addition, the electrode diameter for the maximum mass sensitivity varies with the electrode thickness. Finally, their conclusion was confirmed using a series of coating experiments.

In 2019, Chen et al. continued to investigate the mass sensitivity of the n-m electrode QCM in depth through theoretical calculations and experiments [33]. They calculated the mass sensitivity of 1.4–5.1 QCM, 2.5–5.1 QCM, and 3.5–5.1 QCM separately when the resonant frequency was 10 MHz and the thickness of gold electrodes on both sides was 500 Å and found that the mass sensitivity of 2.5–5.1 QCM was the highest, which indicates that the mass sensitivity of n-m electrode QCM varies with the n and m electrode diameters, and the highest mass sensitivity is achieved when the diameter of the n electrode is close to half of the diameter of the m electrode. Their experiment results are shown in Table 1. The mass sensitivity of the AT-cut 2.5–5.1 QCM at the center of the electrode was calculated to be 3.13 × 10^12^ Hz/kg. In addition, it is worth noting that the mass sensitivity on the n- electrode and m-electrode surfaces is essentially the same in the n-m electrode QCM. Finally, their conclusions were verified in experiments.

## 5. Electrode Thickness and Mass Sensitivity

It is worth noting that most companies providing QCM products do not provide specific values for electrode thickness or simply show a wide range of 400–10,000 or 1000–10,000 Å, which can easily lead to large errors in practice [59,60]. From the analysis in the previous section, it is clear that the electrode thickness can greatly affect the mass sensitivity distribution, especially when the electrode diameter is small. In 2020, Huang et al. first calculated the mass sensitivity of AT-cut 10 MHz QCMs with electrode thicknesses of 10,000, 1500, 1000, 500, and 400 Å. The mass sensitivity of the QCM with the electrode thickness of 10,000 Å is 1.46 and 2.08 times higher than that of QCMs with electrode thicknesses of 1000 and 400 Å, respectively [59]. Then, they conducted coating experiments on m-m electrode QCMs with electrode thicknesses of 500, 1000, and 1500 Å, and compared the mass sensitivity of QCMs with different electrode thicknesses by recording the frequency change before and after plating the gold film with a diameter of 1 mm at the center of the electrode. The quartz wafer and electrode diameters were 8.7 and 4 mm, respectively. Their experiment results are shown in Table 2. The experimental results showed that the mass sensitivity of QCM with 1500 Å thickness was 1.1 and 1.4 times that for 1000 and 500 Å thicknesses, respectively, which also shows that the effect of electrode thickness on the mass sensitivity of the QCM cannot be neglected.

## 6. Electrode Material and Mass Sensitivity

Metals such as gold, silver, and aluminum are commonly used in QCM electrodes. However, researchers generally do not distinguish the mass sensitivity of these QCMs with different electrode materials in practical use [61,62,63,64,65]. This problem was first noted by Huang et al. [32]. They calculated the theoretical absolute mass sensitivity to be 3.84 and 3.11 Hz/ng for gold and silver electrodes, respectively, using an AT-cut 10 MHz QCM with quartz wafer and metal electrode diameters and thicknesses of 8.7 mm, 167 µm, 4 mm, and 1000 Å, respectively. Their theoretical calculations were then confirmed by recording the frequencies of these QCMs before and after attaching a mass film of 500 Å and 1 mm in thickness and diameter, respectively. The experimental results are shown in Table 3. It can be seen that the mass sensitivity of the QCM with gold electrode is significantly higher than that of QCM with silver electrode, which in turn indicates that the mass sensitivity of QCM varies with the electrode material and the mass sensitivity of QCM with different electrode materials is not proportional to the density of the electrode material.

**Figure 9:**
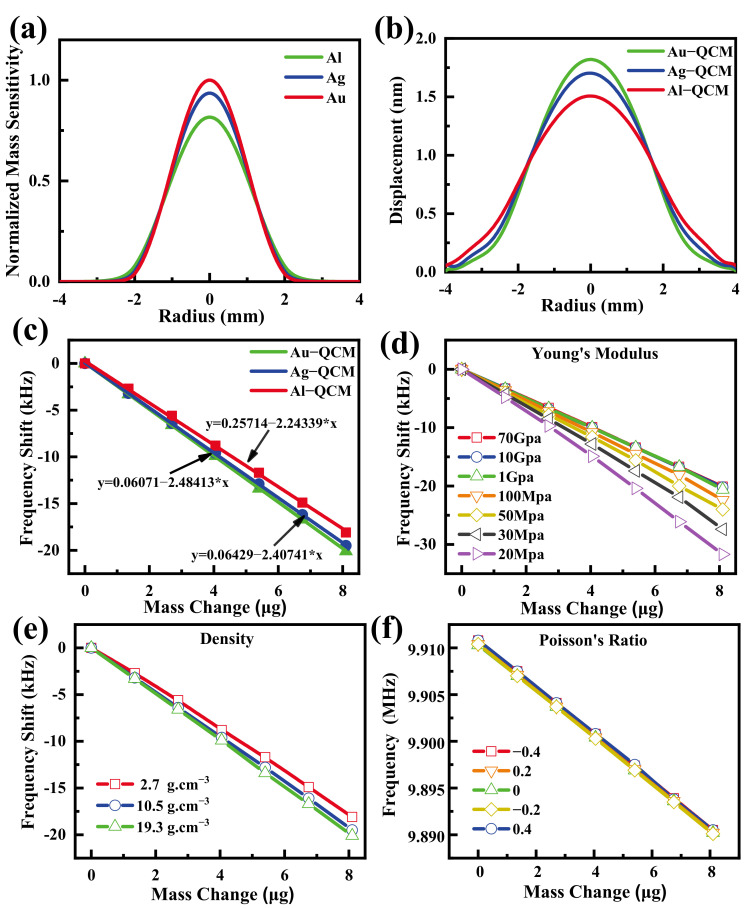
(**a**) The theoretical normalized mass sensitivity, the finite element analysis results of the (**b**) displacement, and (**c**) sensitivity of QCMs with gold, silver, and aluminum electrodes. Finite element analysis of mass sensitivity results at different values of (**d**) Young’s modulus, (**e**) density, and (**f**) Poisson’s ratio [66].

More recently, Chen et al. continued to investigate the effect of electrode material parameters on the mass sensitivity of QCM through theoretical calculations, experimental validation, and finite element analysis methods [66]. They first calculated the mass sensitivity distribution when the electrode materials were gold, silver, and aluminum through the particle displacement amplitude, as shown in Figure 9a, and the authors then innovatively tested the humidity sensitivity of QCM with gold, silver, and aluminum electrodes using graphene oxide (GO) as the humidity-sensitive material and then compared the mass sensitivity of QCM. They then simulated the particle displacement amplitude and mass sensitivity of the QCMs with gold, silver, and aluminum electrodes using the finite element analysis software COMSOL Multiphysics for quartz wafers and metal electrodes and diameters and thicknesses of 8.7 mm, 4 mm, 166 μm, and 100 nm, respectively. It can be seen from Figure 9b,c that the QCM with gold electrode has the largest mass displacement amplitude and mass sensitivity. Finally, from the results of simulating the mass sensitivity of the QCM at different electrode densities, the Young’s modulus and Poisson’s ratio show that it is mainly the density and Young’s modulus of electrode materials that affect the mass sensitivity distribution of the QCM, as shown in Figure 9d–f.

## 7. Operating Frequency and Mass Sensitivity

Improving the mass sensitivity of QCM is one of the aims of many researchers. According to the above analysis, the mass sensitivity of QCM is closely related to its operating frequency. According to the Sauerbrey equation, the mass sensitivity of a QCM operating at a frequency of $nf_{0}$ is $n^{2}$ times that of a QCM operating at a frequency of $f_{0}$. Then in practice, the mass sensitivity of a QCM operating at a frequency of $nf_{0}$ is $n^{3-5}$ times that of a QCM operating at a frequency of $f_{0}$ because the increase in frequency greatly reduces the effective vibration area A in the Sauerbrey equation, which in turn further improves the mass sensitivity of the QCM. Most of the common fundamental frequencies of QCMs were between 5 and 20 MHz. In order to improve the mass sensitivity, Uttenthaler et al. used QCMs with higher frequencies for immunoassays performed with virus-specific monoclonal antibodies and M13 phage and found that the signal-to-noise ratio of QCMs with a fundamental frequency of 56 MHz increased more than 6-fold and the detection limit increased 200-fold, compared to QCMs with a fundamental frequency of 19 MHz [67]. Later, Ogi et al. developed a QCM biosensor with a fundamental resonance frequency of 170 MHz for non-specific staphylococcal protein A with a sensitivity of 15 pg/(cm^2^∗Hz) for human immunoglobulin G (HIgG), which is three orders of magnitude higher than the conventional 5 MHz QCM [68]. Unfortunately, the above mass sensitivity calculations are based on the classical Sauerbrey equation, and there is no precise calculation of the mass sensitivity distribution or direct experimental validation of the mass sensitivity.

**Figure 10:**
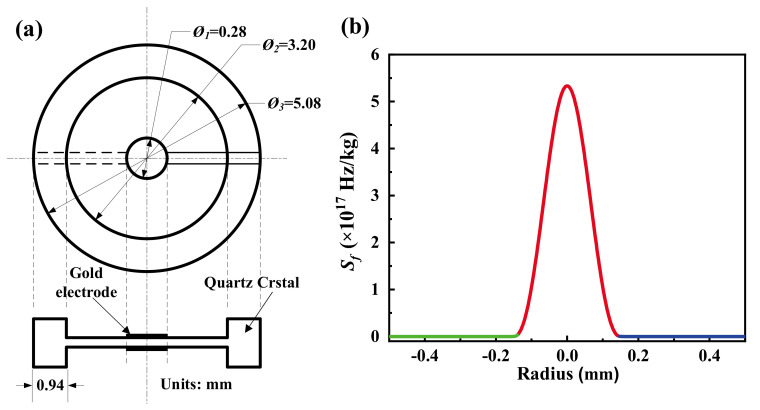
(**a**) The size details of quartz wafer ($\emptyset_{1}$, $\emptyset_{2}$, and $\emptyset_{3}$ are the diameters of the electrode, the etched area, and the quartz crystal plate) and (**b**) mass sensitivity distribution of QCM with a fundamental resonance frequency of 210 MHz based on the inverted mesa structure [69].

In 2019, Chen et al. developed a high-*Q*, 210 MHz, gold m-m electrode QCM using an inverted step structure [69], as shown in Figure 10a. The diameter of the whole QCM and the center etched quartz wafer were 3.2 and 5.08 mm, respectively, and the diameter and thickness of the gold electrode were 0.28 mm and 500 Å, respectively. The mass sensitivity distribution function of this 210 MHz QCM is shown in Figure 10b, while the mass sensitivity at the center of the electrode reaches 5.332 × 10^17^ Hz/kg, which is 5–7 orders of magnitude higher than that of the commonly used 5 or 10 MHz QCMs (which have a mass sensitivity of 10^10^–10^12^ Hz/kg). The average frequency change before and after coating the electrode surface with 1 ng of aluminum film (0.28 mm diameter and 60 Å thickness) at 210 MHz QCM was tested to be 0.192 MHz, verifying its ultra-high mass sensitivity. Meanwhile, the equivalent resistance of the QCM before and after coating was 31.82 and 32.44 Ω, respectively, and the Q value was maintained at 15,781 and 15,597, which is very favorable in practical use.

## 8. Conclusions and Perspectives

This review first introduced the importance, theory, definition, and measuring method of mass sensitivity of the QCM and then reviewed the effects of electrode parameters (electrode shape, electrode diameter, electrode thickness, electrode material, etc.) on the mass sensitivity distribution of QCMs. Finally, the effect of operating frequency on the mass sensitivity of QCMs was analyzed. A summary of the conclusions follows.

(1) Whether in gas phase, liquid droplet, or liquid phase use, the frequency change of QCM is proportional to the equivalent mass sensitivity in the sensing region. Therefore, it is important to accurately calculate and measure the mass sensitivity of QCM.
(2) Uniform mass sensitivity distribution of QCM is beneficial for practical use. The ring electrode QCM can obtain uniform mass sensitivity distribution when satisfying conditions of a suitable (1) ratio of inner and outer diameters; (2) inner diameter value; and (3) electrode thickness (depending on the mass loading factor). These three conditions should be adjusted accordingly when the electrode material is changed.
(3) The thickness of the electrode greatly affects the mass sensitivity distribution of the QCM. Therefore, in practical use, scholars cannot ignore the influence of electrode thickness on the mass sensitivity distribution of QCM, and the company producing QCM should also provide the specific thickness value, which cannot be easily measured by users.
(4) The electrode material also affects the mass sensitivity distribution of QCM. Moreover, the mass sensitivity of QCM with different electrode materials is not proportional to the density of electrode materials.
(5) Increasing the fundamental frequency can significantly improve the mass sensitivity of the QCM due to the electrode effect and the frequency effect.

Although much research has been conducted in the direction of the mass sensitivity of QCM, we believe that the following aspects deserve further attention in the future.

(a) AT-cut quartz crystal is widely used as the basis of QCM. However, the dynamic characteristics of the AT-cut quartz crystal are poor, while the SC-cut type has a theoretical force-frequency coefficient of zero. Therefore, the mass sensitivity of other cut-type quartz crystal microbalances can be investigated in the future to meet the requirements for use in some specific occasions.
(b) The vibration amplitude of the quartz crystal microbalance decays greatly in the liquid phase, while the gallium–lanthanum-based crystal microbalance performs well in liquid phase tests, so the mass sensitivity of other crystal microbalances (LGX) also needs to be investigated in the future.

## Figures and Tables

**Table 1:** The experimental result for the effect of electrode diameter on the QCM mass sensitivity of n-m electrodes [33]. $\;\Delta f_{nm}$ is the frequency change before and after the second plating. $\bar{\Delta f_{nm}}$ and ${\bar{\delta }}_{nm}$ are the average value and the standard deviation of the frequency shift in each subgroup, respectively. $\Delta f_{nm}^{*}$ and $\delta_{nm}$ are the average value and standard deviation of the frequency shift in each group, respectively.

Group	1.4–5.1 QCM		2.5–5.1 QCM		3.5–5.1	
Electrode	n-Electrode	m-Electrode	n-Electrode	m-Electrode	n-Electrode	m-Electrode
$\Delta f_{nm}\;$ (Hz)	2690	2710	2550	2730	2240	2310
	2590	2670	2600	2680	2240	2330
	2380	2640	2700	2760	2110	2290
	2570	2520	2520	2670	2240	2330
	2630	2540	2400	2640	2320	2300
	2480	2610	2640	2620	2270	2270
$\bar{\Delta f_{nm}}\left(\mathrm{Hz}\right)$	2556.67	2615.00	2568.33	2683.33	2236.67	2305.00
${\bar{\delta }}_{nm}$ (Hz)	110.94	73.96	104.39	53.17	69.47422	23.45
$\Delta f_{nm}^{*}\;$ (Hz)	2585.83		2625.83		2270.83	
$\delta_{nm}\left(\mathrm{Hz}\right)$	94.91		99.22		60.97	

**Table 2:** Experimental results on the effect of different electrode thickness on the mass sensitivity of QCM [59].

Thickness	$\Delta f_{e}(\mathrm{Hz})$	$\delta_{t}\;(\mathrm{Hz})$	$\bar{\Delta f_{t}}\;(\mathrm{Hz})$	$C_{QCM}^{*}\;(\mathrm{Hz}/\mathrm{ng})$	$\Delta f_{t}^{*}\;(\mathrm{Hz})$	$E_{t}$
500 Å	1859/2209/2055/2086/2117/2115/2129/2066/	101	2079.5	2.87	2176.4	4.45%
1000 Å	2620/2600/2552/2479/2506/2668/2689/2695/	82.6	2601.1	3.64	2760.3	5.77%
1500 Å	2934/2968/2883/2873/2849/2950/2964/2896/	45.2	2914.6	4.02	3048.4	4.39%

$\Delta f_{t}$ is the frequency change before and after the second plating. $\bar{\Delta f_{t}}$ and $\delta_{t}$ are the average value and the standard deviation of the frequency shift, respectively. $C_{QCM}^{*}$ and $\Delta f_{t}^{*}$ are the equivalent mass sensitivity and theoretical frequency shift, respectively. $E_{t}$ is the deviation between $\bar{\Delta f_{t}}$ and $\Delta f_{t}^{*}$.

**Table 3:** Experimental results on the effect of different electrode materials on the mass sensitivity of QCM [32].

Groups		$f_{m1}\left(Hz\right)\;$	$f_{m2}\left(Hz\right)\;$	$\Delta f_{m}\left(Hz\right)\;$	$\bar{\Delta f_{m}}\;\left(Hz\right)\;$	$\delta_{m}\left(Hz\right)\;$	$\Delta m_{m}\left(ng\right)\;$	$\Delta f_{m}^{*}\left(Hz\right)\;$	*E_m_*
Gold electrode QCM	A	9,961,970	9,959,360	2610	2662	69.79	757.91	2759	3.52%
		9,963,680	9,960,970	2710					
		9,963,990	9,961,360	2630					
		9,962,450	9,959,850	2600					
		9,963,490	9,960,730	2760					
	B	9,962,620	9,961,290	1330	1368	50.20	412.33	1501	8.86%
		9,962,550	9,961,220	1330					
		9,962,080	9,960,730	1350					
		9,962,740	9,961,290	1450					
		9,963,580	9,962,200	1380					
Silver electrode QCM	C	10,000,940	9,998,460	2480	2428	70.90	757.91	2251	7.86%
		10,002,010	9,999,590	2420					
		10,001,480	9,998,960	2520					
		9,999,510	9,997,140	2370					
		9,997,960	9,995,610	2350					
	D	10,001,480	10,000,270	1210	1222	19.24	412.33	1225	0.02%
		10,001,300	10,000,080	1220					
		10,001,650	10,000,450	1200					
		9,999,620	9,998,390	1230					
		9,995,910	9,994,660	1250					

$\Delta f_{m}=f_{m1}-f_{m2}$ is the frequency change before and after the second plating. $\bar{\Delta f_{m}}$ and $\delta_{m}$ are the average value and the standard deviation of the frequency shift, respectively. $\Delta m_{m}$ and $\Delta f_{m}^{*}$ are the mass change and the theoretical frequency shift in the second plating, respectively. *E_m_* is the the deviation between $\bar{\Delta f_{m}}$ and $\Delta f_{m}^{*}$.
